# Artifact reduction of coaxial needles in magnetic resonance imaging-guided abdominal interventions at 1.5 T: a phantom study

**DOI:** 10.1038/s41598-021-02434-5

**Published:** 2021-11-25

**Authors:** Vanessa Franziska Schmidt, Federica Arnone, Olaf Dietrich, Max Seidensticker, Marco Armbruster, Jens Ricke, Philipp Maximilian Kazmierczak

**Affiliations:** 1 Department of Radiology, University Hospital, LMU Munich, Marchioninistr. 15, 81377 Munich, Germany; 2 Department of Radiology Sciences, University of Palermo, Palermo, Italy

**Keywords:** Preclinical research, Imaging techniques

## Abstract

Needle artifacts pose a major limitation for MRI-guided interventions, as they impact the visually perceived needle size and needle-to-target-distance. The objective of this agar liver phantom study was to establish an experimental basis to understand and reduce needle artifact formation during MRI-guided abdominal interventions. Using a vendor-specific prototype fluoroscopic T1-weighted gradient echo sequence with real-time multiplanar acquisition at 1.5 T, the influence of 6 parameters (flip angle, bandwidth, matrix, slice thickness, read-out direction, intervention angle relative to B_0_) on artifact formation of 4 different coaxial MR-compatible coaxial needles (Nitinol, 16G–22G) was investigated. As one parameter was modified, the others remained constant. For each individual parameter variation, 2 independent and blinded readers rated artifact diameters at 2 predefined positions (15 mm distance from the perceived needle tip and at 50% of the needle length). Differences between the experimental subgroups were assessed by Bonferroni-corrected non-parametric tests. Correlations between continuous variables were expressed by the Bravais–Pearson coefficient and interrater reliability was quantified using the intraclass classification coefficient. Needle artifact size increased gradually with increasing flip angles (*p* = 0.002) as well as increasing intervention angles (*p* < 0.001). Artifact diameters differed significantly between the chosen matrix sizes (*p* = 0.002) while modifying bandwidth, readout direction, and slice thickness showed no significant differences. Interrater reliability was high (intraclass correlation coefficient 0.776–0.910). To minimize needle artifacts in MRI-guided abdominal interventions while maintaining optimal visibility of the coaxial needle, we suggest medium-range flip angles and low intervention angles relative to B_0_.

## Introduction

Magnetic resonance imaging (MRI) demonstrates excellent characteristics for image guidance of interstitial interventional procedures, including the lack of ionizing radiation, high soft tissue contrast, and multiplanar needle guidance with simultaneous acquisition of axial, coronal, and sagittal image data sets in near real-time^1–8^. MRI-guided interventions are routinely performed in a broad range of organs, with a primary focus on body regions in which MRI is superior to computed tomography (CT) and ultrasound such as spine, prostate, and liver^1,9–13^. In particular, the combination of fast fluoroscopic T1-weighted gradient echo (GRE) sequences and hepatobiliary contrast agents has even enabled MRI-guided biopsy of small, CT-occult liver lesions with a diameter < 1 cm^14^.

To be MR-compatible and to assure a safe procedure for the patient and the performing radiologist, coaxial needles are made of alloys causing minimal interference with the outer magnetic field, e. g. nickel-titanium (Nickel Titanium Naval Ordnance Laboratory, Nitinol), titanium, glass fiber, or steel^15^. However, needle artifacts presenting as low intensity signal in the region of the coaxial needle cannot be completely excluded and hinder accurate visualization of the needle. In order to successfully perform MRI-guided interventions, a thorough understanding and knowledge of needle artifacts is crucial. The artifact size depends on orientation of the intervention device relative to the B_0_ field (intervention angle), the strength of the static magnetic field, diameter and alloy of the instruments, as well as on pulse sequence type and individual sequence parameters^7,16–18^. Despite being virtually inevitable, needle artifacts pose a major limitation for needle interventions, as they impact the visually perceived size of the needle and distance of the needle tip to the target during the procedure. Therefore, it is essential to limit needle artifacts to a minimum.

Technologic advances in magnet, protocol, coil, biopsy needle, and probe design have made MRI a clinically valuable image-guidance technique. Due to the recent, continuing innovations in targeting software, augmented reality, and compatible devices, it is essential to reassess technical and methodological fundamentals, such as susceptibility artifacts produced by metallic needles, in consideration of these advances. We here present a systematic investigation of artifact behavior of different commercially available MR-conditional Nitinol coaxial needles as a function of the intervention angle and sequence parameter variations in a vendor-specific prototype fluoroscopic T1-weighted GRE sequence with real-time multiplanar acquisition in a dedicated liver phantom at 1.5 Tesla (T). In addition, our study also adds to the field as not only selected but a broad range of clinically relevant parameters was evaluated under standardized and controlled conditions. The aim of the present study was to establish an experimental basis to understand and reduce artifact formation during clinical MRI-guided abdominal interventions in patients.

## Results

Mean values and standard deviations of the artifact diameters of both readers are presented in Table 1.

**Table 1:** Artifact diameter measurements.

Parameter	16G needle	18G needle	20G needle	22G needle	Mean
**Flip angle (°)**					
5	12.1 ± 0.1	11.9 ± 0.1	6.7 ± 0.2	5.8 ± 0.2	9.1 ± 2.1
10	12.1 ± 0.0	11.9 ± 0.0	6.7 ± 0.3	5.8 ± 0.3	9.1 ± 1.8
15	12.1 ± 0.3	11.9 ± 0.1	6.5 ± 0.2	5.8 ± 0.3	8.9 ± 1.9
20	12.1 ± 0.1	11.8 ± 0.2	6.7 ± 0.2	6.5 ± 0.3	9.3 ± 2.5
25	12.3 ± 0.4	11.7 ± 0.0	7.2 ± 0.1	6.9 ± 0.2	9.5 ± 2.3
30	12.3 ± 0.2	11.7 ± 0.3	7.4 ± 0.0	7.0 ± 0.1	9.6 ± 2.5
45	12.3 ± 0.3	11.8 ± 0.2	7.7 ± 0.1	7.1 ± 0.1	10.1 ± 2.9
60	12.5 ± 0.2	11.9 ± 0.2	7.8 ± 0.0	7.1 ± 0.2	10.5 ± 2.4
75	16.0 ± 0.8	15.2 ± 0.3	9.2 ± 0.2	8.3 ± 0.2	12.2 ± 2.9
90	16.1 ± 0.6	15.4 ± 0.4	9.8 ± 0.3	8.5 ± 0.3	13.3 ± 3.0
**Bandwidth (Hz/pixel)**					
300	12.5 ± 0.1	11.7 ± 0.1	8.9 ± 0.2	6.7 ± 0.3	9.8 ± 2.6
400	12.6 ± 0.1	12.0 ± 0.2	9.0 ± 0.2	5.9 ± 0.1	9.8 ± 2.8
500	12.5 ± 0.3	11.8 ± 0.1	8.9 ± 0.1	5.7 ± 0.2	9.7 ± 2.9
600	12.2 ± 0.2	12.0 ± 0.2	8.4 ± 0.3	5.6 ± 0.2	9.5 ± 2.9
700	12.7 ± 0.0	10.8 ± 0.9	8.5 ± 0.0	5.9 ± 0.2	9.5 ± 2.8
**Matrix**					
96 × 96	12.7 ± 1.4	11.6 ± 1.2	8.6 ± 0.8	7.9 ± 0.4	10.2 ± 2.4
128 × 128	12.3 ± 0.3	10.5 ± 1.0	6.9 ± 0.7	5.8 ± 0.2	8.6 ± 2.9
192 × 192	11.4 ± 0.8	09.6 ± 0.2	6.8 ± 0.7	6.1 ± 0.4	8.4 ± 2.5
256 × 256	10.9 ± 1.4	09.5 ± 0.7	7.1 ± 0.1	6.2 ± 0.3	8.4 ± 2.2
**Slice thickness (mm)**					
7	11.8 ± 1.5	11.3 ± 0.9	6.8 ± 1.1	5.4 ± 0.4	8.8 ± 3.2
10	12.1 ± 0.9	11.1 ± 0.5	6.5 ± 0.2	5.5 ± 0.4	8.8 ± 3.2
13	12.3 ± 0.7	10.2 ± 0.5	6.7 ± 0.5	5.2 ± 0.2	8.6 ± 3.0
16	11.8 ± 0.7	10.6 ± 0.4	7.4 ± 0.4	5.2 ± 0.7	8.7 ± 2.8
**Read-out direction**					
A > > P	12.6 ± 0.5	10.1 ± 0.6	6.8 ± 0.5	5.5 ± 0.2	8.8 ± 3.1
R > > L	12.5 ± 0.7	10.3 ± 0.2	6.6 ± 0.3	5.5 ± 0.3	8.7 ± 2.6
**Intervention angle (°)**					
0	5.8 ± 0.2	6.3 ± 1.0	5.0 ± 1.2	4.0 ± 0.7	5.2 ± 1.3
15	6.7 ± 0.7	6.7 ± 0.1	5.4 ± 0.5	3.9 ± 0.4	5.7 ± 1.4
30	8.3 ± 1.1	6.8 ± 1.0	5.5 ± 0.4	5.2 ± 0.1	6.5 ± 1.5
45	10.8 ± 2.2	9.3 ± 0.9	7.3 ± 0.7	5.7 ± 0.7	8.2 ± 2.5
60	12.6 ± 0.8	11.6 ± 0.9	8.5 ± 0.3	6.8 ± 0.6	9.8 ± 2.6
75	15.2 ± 0.6	13.0 ± 0.1	9.3 ± 0.1	7.6 ± 0.1	11.3 ± 3.2
90	14.5 ± 1.2	13.3 ± 0.4	9.0 ± 0.4	7.5 ± 0.9	11.1 ± 3.2

Measured artifact diameters (in mm) at various sequence parameters, averaged over both readers. Values presented as means ± SD.

*SD* standard deviation.

### Flip angle

**Figure 1:**
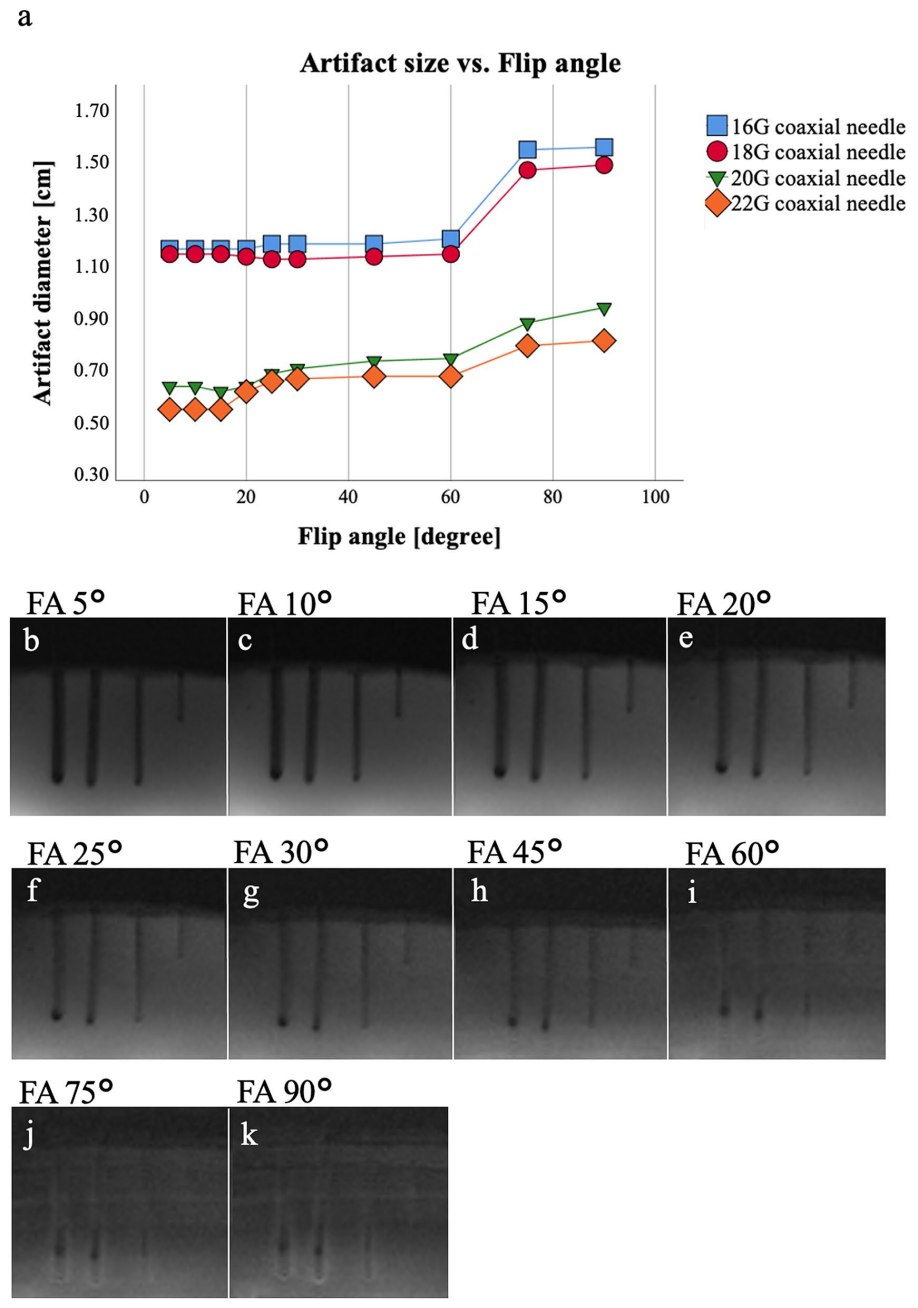
Scan series 1. Mean values (of both readers) of the measured artifact diameters (**a**) of the 16G, 18G, 20G, 22G needles with different FA of the T1-weighted GRE sequence. Note the positive correlation of artifact diameter and FA (*p* ≤ 0.05) for 16G (*r* = 0.859), 18G (*r* = 0.816), 20G (*r* = 0.965), and 22G (*r* = 0.950) MR-compatible needles, in (**b**–**k**) the visual correlate is shown. Note the hyperintense rim around the central hypointense needle artifact occurring with FA > 45° representing as an alteration of the image contrast due to the needle. FA = flip angle, G = gauge.

Artifact size increased gradually with increasing FA for each needle diameter (16G: 12.1–16.1 mm, 18G: 11.9—15.4 mm, 20G: 6.7–9.8 mm, 22G: 5.8–8.5 mm; *p* = 0.002). Pairwise comparison did not show any significant difference between stepwise increased parameters. However, multiple testing revealed significant differences between 9 pairs of FA. For all 4 needles, significant and strong positive correlations between FA and artifact diameter (*r* between 0.816 and 0.965; *p* < 0.01) (Fig. 1) were observed. In addition to the central hypointense needle artifact, a hyperintense peripheral rim was observed at FA > 45°, which was included in the measurements.

### Bandwidth

**Figure 2:**
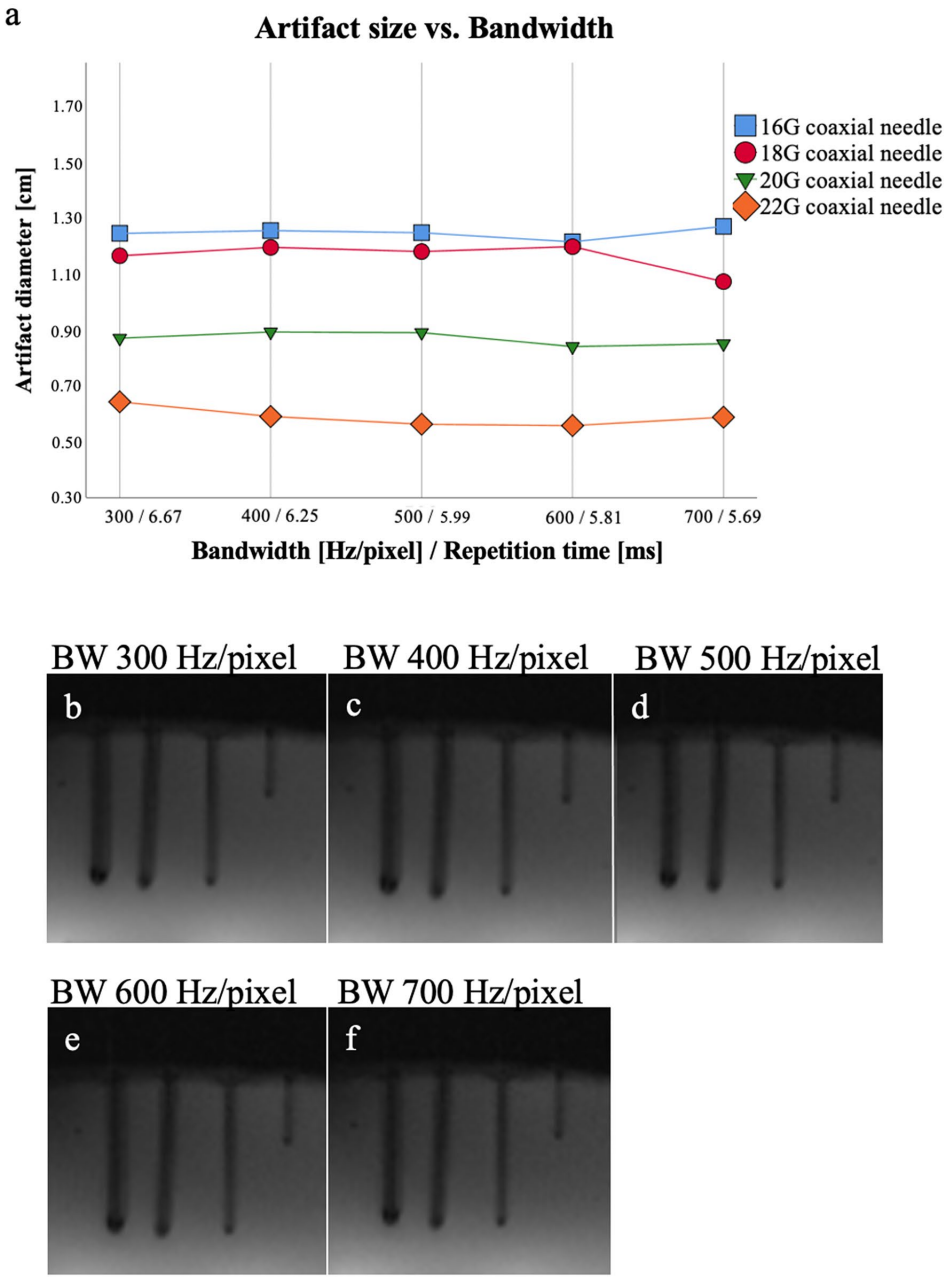
Scan series 2. Mean values (of both readers) of the measured artifact diameters (**a**) of the 16G, 18G, 20G, 22G needles with different BWs of the T1-weighted GRE sequence. There was no significant correlation of artifact diameter with BW for 16G (*r* = 0.300), 18G (*r* = − 0.100), 20G (*r* = − 0.600), and 22G (*r* = − 0.700) MR-compatible needle, in (**b**–**f**) the visual correlate is shown. BW = bandwidth, Hz = Hertz, G = gauge.

Modifying the receiver BW, we found no significant difference in artifact diameter for any of the different needles (16G: 12.2–12.7 mm, 18G: 10.8–12.0 mm, 20G: 8.4–9.0 mm, 22G: 5.6–6.7 mm; *p* = 0.304) and also no significant correlation (− 0.70 < *r* < 0.62, *p* > 0.19) (Fig. 2) between the artifact diameter and the BW.

### Matrix

**Figure 3:**
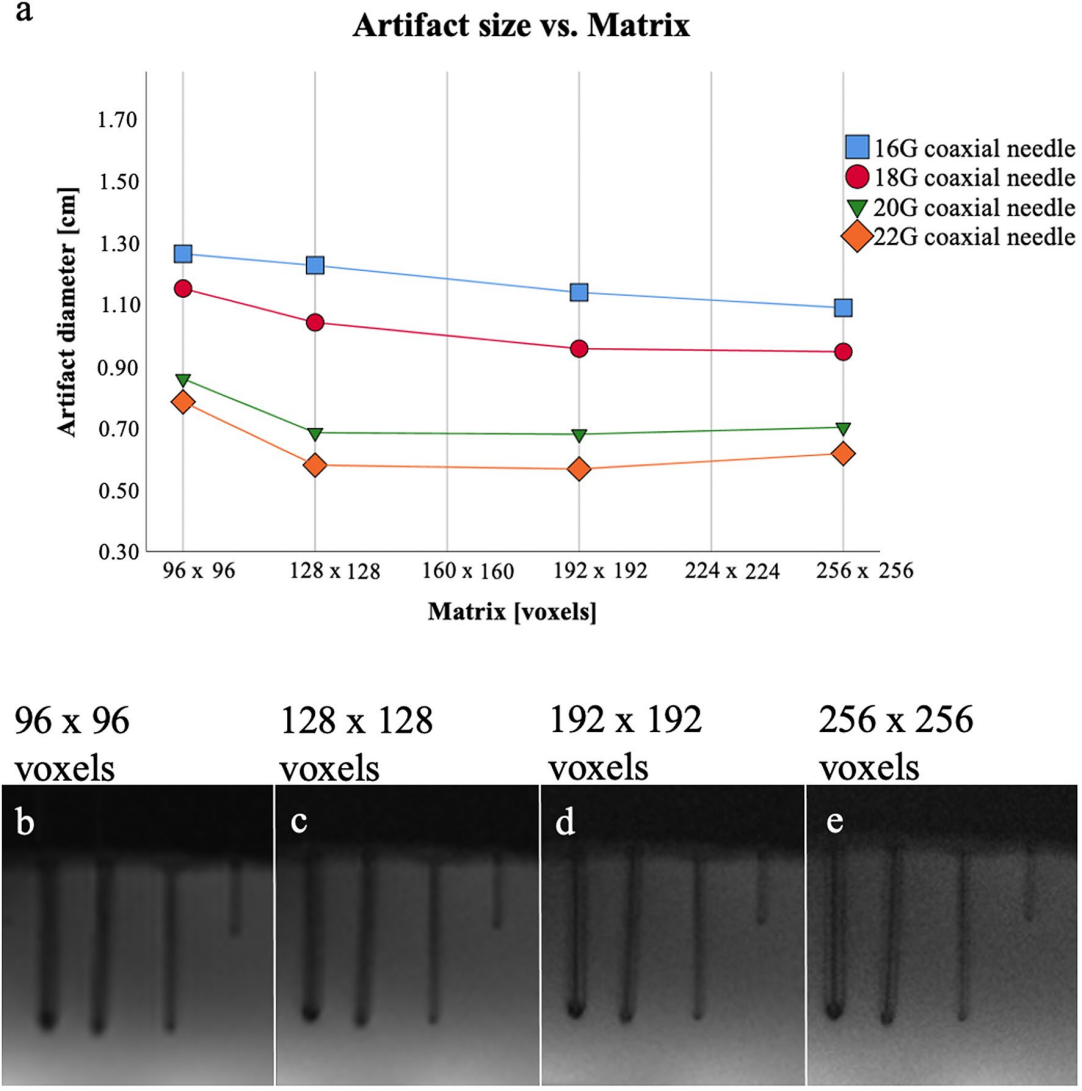
Scan series 3. Mean values (of both readers) of the measured artifact diameters (**a**) of the 16G, 18G, 20G, 22G needles with different matrix sizes of the T1-weighted GRE sequence. There was a negative correlation (*p* < 0.005) of artifact diameter with matrix for 16G (*r* = − 0.993) needles, but no significant correlation for 18G (*r* = − 0.905), 20G (*r* = − 0.611), and 22G (*r* = − 0.549) needles. In (**b**–**e**) the visual correlate is shown. Note the increasing differentiation between actual needle and artifact at larger matrices. G = gauge.

Artifact diameters differed significantly between the 4 matrix sizes for each needle diameter (16G: 10.9–12.7 mm, 18G: 9.5–11.6 mm, 20G: 6.8–8.6 mm, 22G: 5.8–7.9 mm; *p* = 0.002); however, pairwise comparisons did not demonstrate any significant differences excluding between the matrix sizes 96 × 96 and 192 × 192 (*p* = 0.004) as well as between 96 × 96 and 256 × 256 (*p* = 0.006). A significant negative correlation was found between the mean artifact diameter of both readers and the matrix for the 16G needle (*r* = − 0.993, *p* = 0.007). For the other needle sizes, there was no significant correlation (Fig. 3) between the artifact diameter and the matrix. In addition, increasing differentiation between the display of the “actual” needle and the surrounding artifact at larger matrices could be observed.

### Slice thickness

Modifying slice thickness, we found no significant difference in artifact diameter for each needle size (16G: 11.8–12.3 mm, 18G: 10.2–11.3 mm, 20G: 6.5–7.4 mm, 20G: 5.2–5.5 mm; *p* = 0.191) and no significant correlation between artifact diameter and slice thickness for any of the needles either (− 0.787 < *r* < 0.662; *p* > 0.21).

### Readout direction

For the 2 varying readout directions (right > left, anterior > posterior), no significant differences in artifact diameter were found according to Wilcoxon signed-rank test (*p* = 0.623) with generally similar artifact diameters for both read-out directions (Table 1).

### Intervention angle

**Figure 4:**
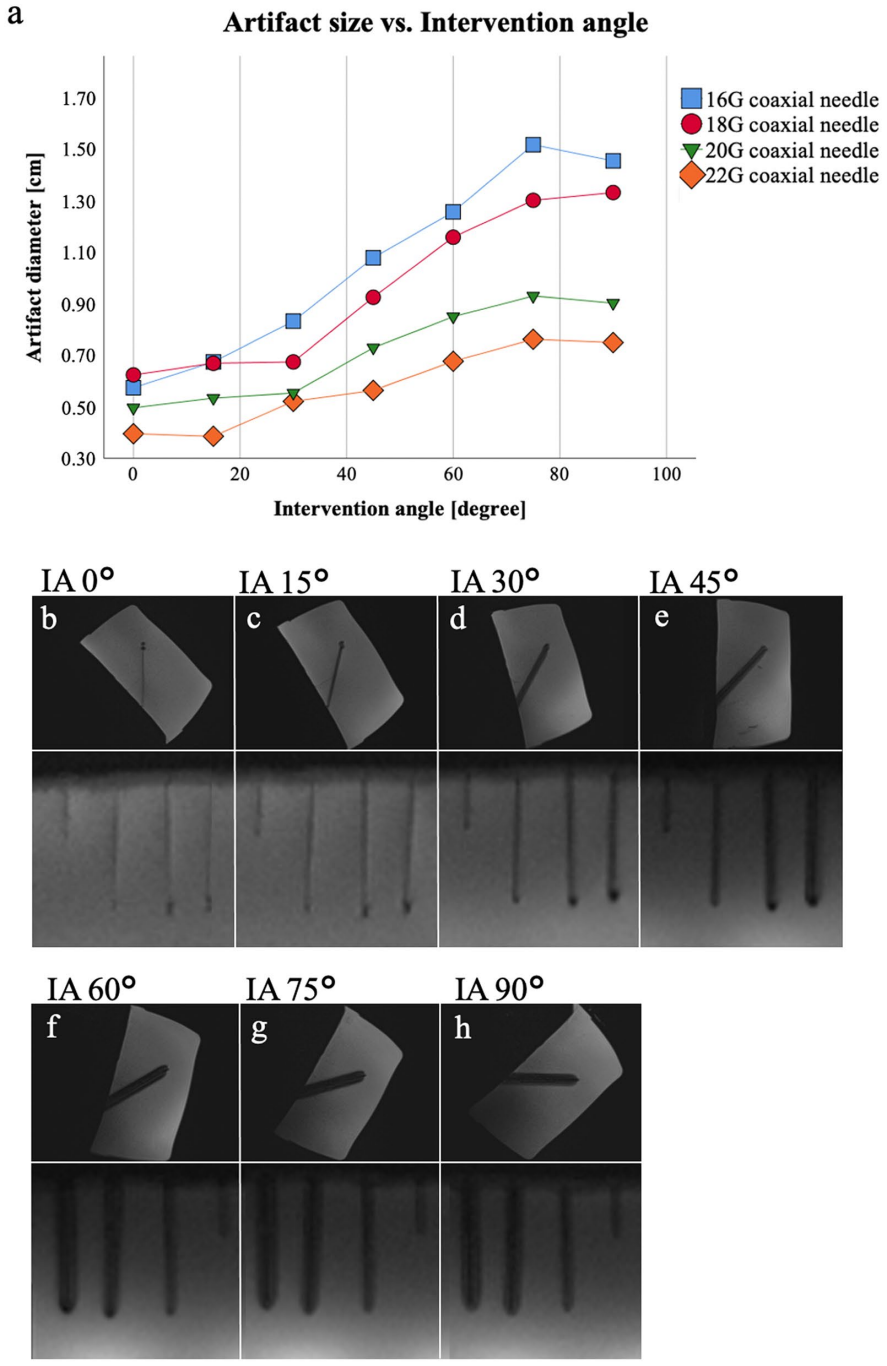
Scan series 6. Mean values (of both readers) of the measured artifact diameters (**a**) of the 16G, 18G, 20G, 22G needles with different intervention angles of the T1-weighted GRE sequence. Note the positive correlation of artifact diameter with intervention angle (*p* ≤ 0.05) for 16G (*r* = 0.980), 18G (*r* = 0.966), 20G (*r* = 0.960), and 22G (*r* = 0.972) MR-compatible needles; in (**b**–**h**) the visual correlate is shown. The upper row provides demonstration purposes of the used angulation. The lower row shows the sequence images used for artifact measurements. Note the ball-like tip artifact occurring at small intervention angle (0° and 15°). IA = intervention angle, G = gauge.

Artifact size increased gradually with increasing intervention angles for each needle diameter (16G: 5.8–15.2 mm, 18G: 6.3–13.3 mm, 20G: 5.0–9.3 mm, 22G: 3.9–7.6 mm; *p* < 0.001). Pairwise comparisons did not show any significance between the stepwise changes, exemplarily from 0° to 15° (*p* = 1.000) or from 60° to 75° (*p* = 1.000). Multiple testing revealed significant differences of the artifact diameter for 7 pairs of intervention angles. For all 4 needles, we observed a significant positive correlation (*r* between 0.960 and 0.980; *p* ≤ 0.001) with strong effect strength, which is shown in Fig. 4. In addition, a ball-like tip artifact was observed at small intervention angles (0° and 15°).

### Needle diameter

**Figure 5:**
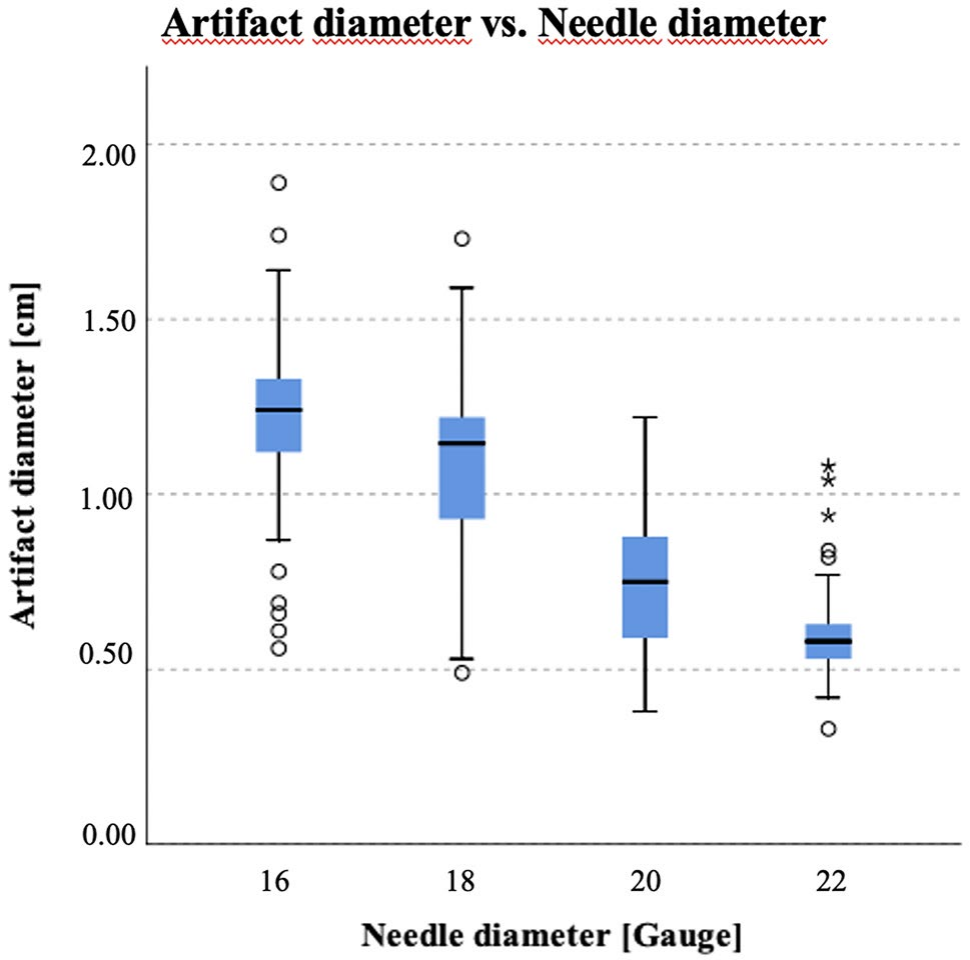
Boxplot of artifact size depending on needle diameter. Medians (of both readers) of all measured artifact diameters of all modified parameters separated in 16G, 18G, 20G, 22G needles. Kruskal–Wallis test demonstrated a significant difference of artifact diameter (*p* < 0.001) for the 4 sizes of MR-compatible needles.

The 4 needle diameters (16–22G) showed significant differences (*p* > 0.001) in artifact diameter (Fig. 5), which also proved to be significant (*p* < 0.05) for each pairwise comparison, except between 16 and 18G (*p* = 0.064).

### Interrater reliability

For all 4 needles, the intraclass correlation coefficients were between 0.776 and 0.910 (*p* < 0.001) indicating excellent interrater reliability (Table 2).

**Table 2:** Interrater reliability.

MR-compatible needle	ICC value	*p*-value	95% confidence interval
16G	0.858	*p* < 0.001	0.744–0.923
20G	0.910	*p* < 0.001	0.835–0.952
18G	0.848	*p* < 0.001	0.729–0.918
20G	0.776	*p* < 0.001	0.611–0.876

Intraclass correlation coefficient (ICC) of 2 blinded reviewers: It is shown the interrater reliability of the measured artifact diameters for each needle diameter.

## Discussion

In this phantom study, we investigated the influence of different sequence parameters of a vendor-specific prototype fluoroscopic T1-weighted GRE sequence with near real-time multiplanar acquisition and of the intervention angle on artifact behavior of commercially available, MR-compatible coaxial needles at 1.5 T. Needle artifacts in MRI are caused by several physical processes, but in particular by inhomogeneities of the static magnetic field (B_0_) experienced by the nuclei. The needle-induced B_0_ inhomogeneity stems from the geometric characteristics and the magnetic susceptibility of the object being imaged. These static field errors cause distortions of the spatial geometry, as well as intra-voxel dephasing. The latter is caused by local disturbances of B_0_ within a voxel resulting in severe intensity losses due to uncompensated intravoxel phase dispersion (T2* effect), which is particularly relevant for GRE sequences^18,19^. Other causes for needle artifacts are radio-frequency effects such as B1 enhancement^20^. We aimed to investigate these processes (summarized by the term “needle artifacts” in this study) in whole regarding their relevance for MR-guided abdominal procedures depending on easily modifiable sequence parameters and intervention angle. Our study adds to the field as it provides a comprehensive, systematic basis for future investigation and potential reduction of needle artifacts during clinical MR-guided abdominal interventions.

Increasing artifact diameters were observed with increasing FA. The hyperintense peripheral rim around the central hypointense needle artifact occurring at high FA (> 45°) was included in the measurement. It extends the area of potential misinterpretation of needle position and should be considered when choosing a high FA for T1-weighted GRE sequences. Another workgroup already investigated needle artifacts by modifying the FA in T1-weighted GRE sequences^14^. In line with our results, they did not report any relevant change of the artifact diameter in a stepwise comparison of different artifact diameters for FA < 45°. Only when comparing FA 45° to 55° and 55° to 65°, a significant increase in artifact diameter was demonstrated, while including the hyperintense zone in the measurement. Overall, the results are in accordance with our observation that optimal artifact diameters are found with FA < 45°^14^. Another aspect to consider is the signal-to-noise ratio (SNR) decreasing with increasing FA above the Ernst angle (α_E_) in GRE sequences^21^. It is known that the MR signal is maximized at α_E_, which can be calculated from the equation α_E_ = arccos (exp(− TR/T1)). Using the presented real-time, T1-weighted GRE sequence at 1.5 T with a short TR of 5.3 ms, this would result in an α_E_ of 10.7°, calculated for healthy liver parenchyma showing T1 relaxation time of ~ 300 ms after 10–20 min of Gd-EOB-DTPA administration^22,23^. In the case of cirrhotic or functionally impaired liver parenchyma, longer T1 and thus an even lower α_E_ would have to be expected. In general, the α_E_ is low in combination with the short TR used in our study, so that high as well as medium FAs may not lead to optimal SNR.

The modification of the BW showed no significant influence on the artifact diameter though BW has been mentioned in the literature as one of the crucial modification factors to minimize needle artifacts^24^. The theory behind this is, that by a metallic object of a given size and susceptibility, the Larmor frequencies of the hydrogen protons are altered to a certain amount^25^. Reducing the Hz/pixel ratio increases the number of pixels affected by the variance in frequencies and thus increases the size of the susceptibility artifact. Overall, it can be concluded that for MR-guided interventions with T1-weighted GRE sequences, BW variations did not show any significant effect on needle artifact size in the investigated range, which might have been too small to reveal significant differences. However, the BW range was chosen according to standard BW used in clinical routine.

For the various matrix sizes, a significant difference in artifact diameter was observed. In addition, a negative correlation between artifact diameter and selected matrix was found for the 16G needle. The other needles also showed smaller artifact diameters at higher matrix sizes, but the effect was not strong enough to be significant for the matrix sizes evaluated in this work. An increased matrix not only reduces the artifact diameter but also optimizes the image quality due to decreased voxel volume producing MR images that may show more small details. The matrix determines the spatial resolution and is therefore a quality feature of the acquired image data^26^. The improved differentiation of the actual needle and artifact shown in our scan series allows more exact biopsy of target lesions. These advantages of a smaller artifact diameter and a higher spatial resolution are offset by an increase in acquisition time, which is the crucial point that the matrix cannot be set as high as possible^14^.

As the matrix yields an influence on the voxel size, it is also impacted by slice thickness. If the slice thickness decreases, which is equivalent to a reduction of the voxel size, the field inhomogeneity within each individual voxel decreases. This results in lower artifact dependence and generates smaller needle artifacts^26,27^. In the present study, we could not observe any significant differences between the selected slice thicknesses with regard to artifact size. However, it must be noted that the differences in the extent of the change of the artifact diameter may have been too small.

The comparison of the readout directions (right > left, anterior > posterior) at an intervention angle of 45° revealed no significant differences with regard to artifact diameters. Our results are in accordance with previous studies. Lewin et al. compared parallel and perpendicular readout directions at the intervention angle of 90° and found no significant differences for GRE sequences; this is in contrast to spin echo (SE) and turbo spin echo (TSE) sequences, in which artifacts were more pronounced when the readout direction was perpendicular to the needle shafts^28^. Similarly, Frahm et al. reported no provable effect swapping the phase-encoding and frequency-encoding axes on GRE sequences at the intervention angle of either 0° or 90°, respectively^26^.

The intervention angle is closely related to the size of the resulting artifact^18,29,30^. In accordance, our results also showed the positive correlation of artifact size with increasing intervention angle relative to the B_0_ field. Using low susceptibility materials is advantageous as these may be used at higher angles. Frahm et al. investigated the relationship between magnetic field strength and intervention angle^26^. They demonstrated that at lower field strengths (0.2 vs. 1.5 T), the needle artifact growths less with increasing angle. At high field strengths, the artifact correlates closely with the increasing angle relative to the B_0_ field^26^. In addition, a ball-like tip artifact at the tip of the needle was observed at the low intervention angles (0°–10°). As described by the working group of Liu et al., the magnetic field is most strongly influenced in the area of the needle tip^31^. This is particularly noticeable in needles with lower magnetic susceptibility, such as carbon fiber or titanium, compared to chromium, cobalt, or nickel, respectively. This three-dimensional tip artifact is significantly larger than the rather small artifact along the needle body at small angles and extends in all directions^14,32^. As already shown in previous works^14,29^, the influence of the needle diameter can be observed in each individual parameter tested and also in the evaluation of all measurements performed: Larger needles show increased artifact sizes, but the most significant difference was observed between 18 and 20G.

With regard to clinical practice, we recommend the lowest reasonably achievable intervention angles relative to the B_0_ field, even if repositioning of the patient becomes necessary. Furthermore, we suggest medium-range FA (30°–45°) to minimize the hypointense needle artifact and to avoid the hyperintense rim in the artifact periphery, which exclusively occurred at high FA in our scan series.

We acknowledge several limitations to the present study. First, imaging was performed under standardized laboratory conditions, as the phantom was fixed, motionless, and provided a high background signal intensity that facilitated the depiction of signal voids. In vivo, lower artifact contrast must be expected, as well as organ movement due to diaphragmal excursions during breathing. In addition, the clinical employment of higher FA may be limited by high specific absorption rates (SAR) and strong signal saturation, potentially hindering visibility of the target structures. Second, the evaluation of the artifact diameter was performed by manual measurements. Automatic artifact measurements would minimize the potential impact of the reader. However, high interreader agreement was observed in the present study. Third, we only investigated a single alloy (Nitinol). Different alloys may yield different artifact properties and should be investigated in further studies. Fourth, the phantom study was performed at a single field strength (1.5 T). For other organ regions, such as the prostate gland, higher field strengths (3 T) will need to be investigated. Fifth, when modifying the parameters in our evaluation, we tried to avoid changing another parameter at same time in order to not have any additional confounding variables. However, BW was not modified separately but only coupled to TR due to the fact that the minimum TR was systematically chosen in our experimental setting. One consequence of that choice could actually be misleading as the SNR increases with increasing TR. In addition, in a static phantom, images do not suffer from additional motion artifact with increasing image acquisition time (with increasing TR). Sixth, low artifact size in some instances does not necessarily mean that the “true” position of the needle is better known, as it is extremely difficult to be sure about the exact needle position from the real-time MRI visualization. Further studies are needed here, e.g., using a coordinate/navigator system to determine and verify the actual needle position and particularly the exact position of the needle tip in longitudinal direction along the needle axis. However, several issues remain to be resolved here, such as integration of, e.g., a coordinate system into the agar phantom and minimizing errors of measurement using the 2D T1 GRE sequence, taking into consideration that the distance between the grid and the needle will never be zero. Yamada et al. recently made an attempt in this direction, as they applied real-time US imaging fused with reformatted static MR images and coordinate registration for needle guidance during MR-guided percutaneous tumor ablations, with a mean targeting error of 1.6 ± 0.6 mm^33^. However, to date there is a lack of similar studies using real-time MR sequences. Seventh, we performed 2-dimensional (D) artifact measurements. However, it is acknowledged that in 3D image-guided interventions, needle artifact volume may be a relevant additional parameter. Though, depending on the sequence acquisition, such an assessment may be not adequately feasible, and several recent studies were also conducted in 2D^34,35^. Nevertheless, artifact volume and therefore possible differences to our measurement method should be investigated in future studies. Eighth, individually iterated the optimal parameters in our scan series, the combination of these settings and their interaction with regard to artifact formation needs to be investigated in further studies and in vivo.

### Conclusion

Low intervention angles relative to the B_0_ field and medium-range FAs minimized the needle artifacts while maintaining optimal visualization of the intervention needle in this phantom study at 1.5 T.

## Methods

This phantom study did not require institutional review board approval.

### Image acquisition

MRI was performed on a closed-bore clinical 1.5 T MRI scanner (Magnetom Aera, Siemens Healthineers, Erlangen, Germany) with a short open bore design (system length cover-to-cover 145 cm, bore diameter 70 cm). The gradient system had a maximum gradient strength of 45 mT/m and a slew rate of 200 T/m/s. The MRI protocol was based on a prototype real-time fluoroscopic T1-weighted GRE sequence (Siemens WIP package ASP 1075G “Needle AutoAlign”, Siemens Healthineers, Erlangen, Germany). This prototype package allows for visual real-time update and interactive graphical modification of the slice geometry during imaging. A 6-channel body coil (Siemens Healthineers, Erlangen, Germany) with a weight of 1.4 kg (322 mm × 533 mm × 70 mm) was used as receive coil.

### Agar phantom

**Figure 6:**
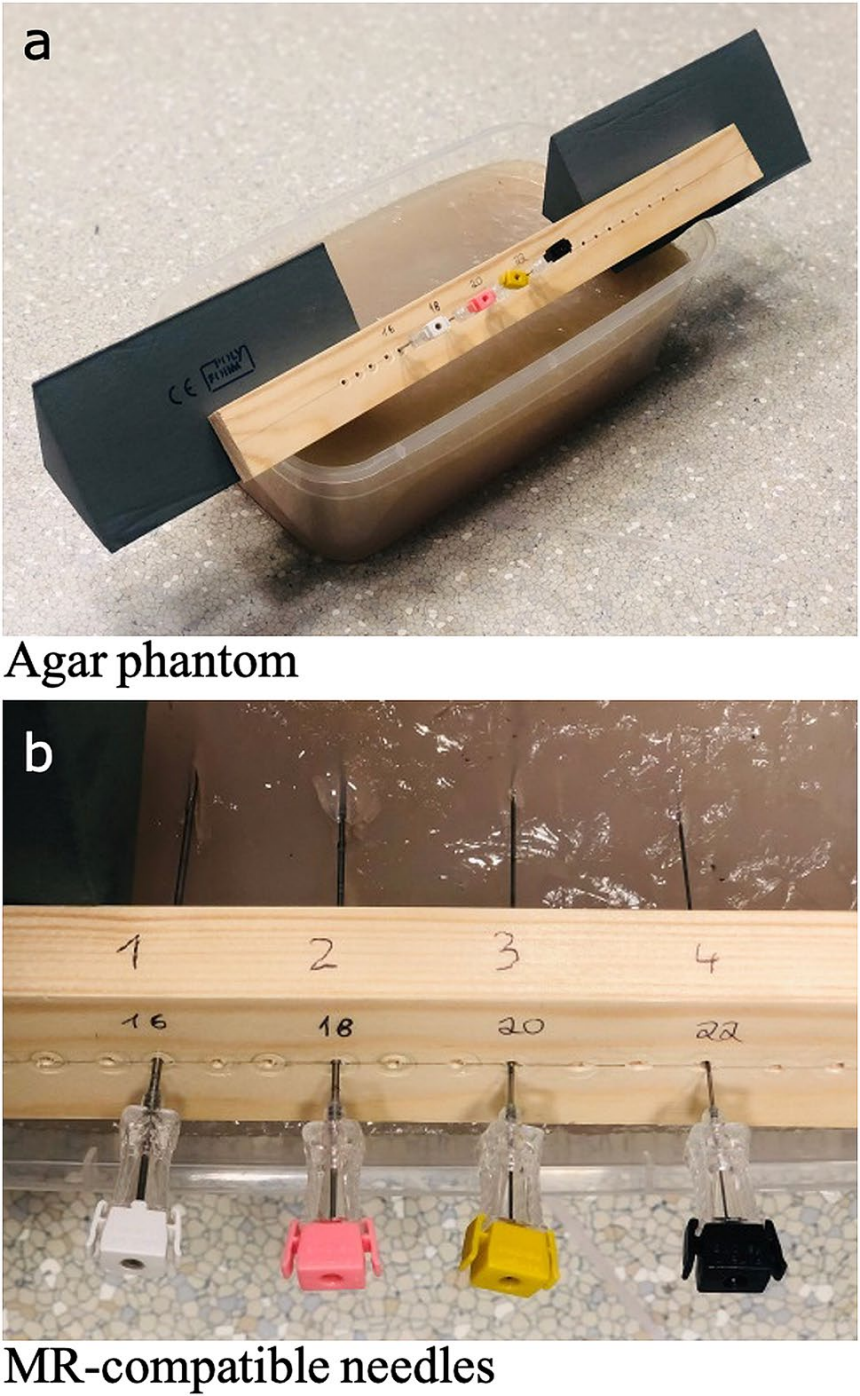
Experimental setup. Agar phantom with needle holder device (**a**): The phantom was composed of water, agar–agar, and sodium chloride and cured in a polyvinyl chloride container. Coaxial MR-compatible needles with different diameters (**b**) were introduced in a needle holder device firmly attached to the polyvinyl chloride container at a 45° angle. To evaluate the individual intervention angles to the B_0_ field, the phantom was tilted towards the B_0_ field for the intervention angles 30°, 15° and 0° and in the opposite direction for the angles 60°, 75° and 90°.

The dedicated liver phantom was created by mixing 5 L of water with 100 g sodium chloride and 100 g agar–agar. The suspension was poured into a polyvinyl chloride (PVC) container and the subsequent curing process occupied 24 h at room temperature (21 °C). The agar phantom had a viscosity and signal intensity similar to that of the human liver parenchyma, as previously described^36,37^. The course of the needle could easily be followed from the outside due to the corresponding transparency. The repeated production of the phantom was not necessary as the complete scan series could be performed using the same model, assuring comparability of the results. An artifact-free wooden needle holder device with predetermined small openings was used for the exact and parallel positioning of the intervention needles in the B_0_ field and held the needles in a stable position. The experimental setup is shown in Fig. 6.

### MR-compatible intervention needles

We investigated 4 different commercially available MR-compatible coaxial Nitinol needles (ITP Innovative Tomography Products GmbH, Bochum, Germany) sized 16G (outer diameter 1.60 mm, length 140 mm, trocar cut), 18G (outer diameter 1.25 mm, length 150 mm, standardized facet cut), 20G (outer diameter 0.90 mm, length 150 mm, standardized facet cut), and 22G (outer diameter 0.70 mm, length 100 mm, standardized facet cut).

### Scan series

The phantom was positioned in the isocenter of the XZ plane using the light visor of the magnetic resonance tomograph. The influence of the following parameters on artifact formation was investigated: flip angle, receiver bandwidth, matrix, slice thickness, read-out direction, and intervention angle (i. e. the needle angle relative to the B_0_ field). As one parameter was modified, the others remained constant in the following predefined setting: The matrix was fixed to 128 × 128 voxels, which was a compromise between acquisition time and spatial resolution. Echo time (TE) and repetition time (TR), which yield an influence on acquisition time, were set to a minimum (TE 2.35 ms, TR 5.3 ms) as fixed parameters, resulting in an acquisition time of 0.68 s per plane. The field of view (FOV) was uniformly set to 300 × 300 mm^2^. Image acquisition was performed at a 45° angle relative to the B_0_ field as fixed intervention angle. The predefined setting was 15° for the flip angle (FA), 500 Hz/pixel for the bandwidth (BW), and 10 mm for the slice thickness, respectively. The fixed read-out direction was anterior to posterior. A detailed overview of these fixed parameters of predefined setting is provided in Table 3.

**Table 3:** Default settings.

Fixed parameter	Value
FOV (mm^2^)	300 × 300
Matrix (voxels)	128 × 128
Slice thickness (mm)	10
Flip angle (°)	15
Echo time (ms)	2.35
Repetition time (ms)	5.3
Bandwidth (Hz/pixel)	500
Read-out direction	A > > P
Intervention angle (°)	45
Phase oversampling	0
Acquisition time (ms)	682

While one parameter was modified, all others remained unchanged in a predefined setting.

Starting with these default settings, each of the parameters mentioned above was modified, as described in detail in Table 4, resulting in acquisition times of 0.68–1.53 s per plane. For each parameter modification, the T1-weighted real-time sequence was performed in the same manner. The needle holder was attached to the phantom at a 45° (default setting) angle to the orientation of the B_0_ field extending longitudinally into the bore. To evaluate the individual intervention angles to the B_0_ field, the whole phantom was tilted towards the B_0_ field for the intervention angles 30°, 15°, and 0°, as well as in the opposite direction for the angles 60°, 75°, and 90°, respectively. Prior to the start of the fluoroscopic T1-weighted GRE sequence, the correct angle (accepted deviation of 0–2°) between the needles and the B_0_ field and the positioning in the isocenter of the MR imager was verified by test sequences.

**Table 4:** Flowchart.

Scan series	Values	Coupled TR (ms)	
Flip angle (°)	1	5	
	2	10	
	3	15	
	4	20	
	5	25	
	6	30	
	7	45	
	8	60	
	9	75	
	10	90	
Bandwidth (Hz/pixel)	1	300	6.67
	2	400	6.25
	3	500	5.99
	4	600	5.81
	5	700	5.69
Matrix (voxels)	1	96 × 96	
	2	128 × 128	
	3	192 × 192	
	4	256 × 256	
Slice thickness (mm)	1	7	
	2	10	
	3	13	
	4	16	
Readout direction	1	A > > P	
	2	R > > L	
Intervention angle (°)	1	0	
	2	15	
	3	30	
	4	45	
	5	60	
	6	75	
	7	90	

Scan series of study profile.

Systematic and sequential modification of the intervention angle and of the technical parameters of T1-weighted GRE sequences.

*TR* repetition time, *Hz* Hertz, *A* anterior, *P* posterior, *R* right, *L* left.

### Artifact diameter measurement

For image acquisition and evaluation of the artifact diameter, the software syngo Studio VB36E (Siemens Healthineers, Erlangen, Germany) was used. To ensure comparability of the individual artifact diameter, artifact diameter was measured in standardized planes at 2 defined positions: in 15 mm distance from the perceived needle tip and at 50% of the total needle length. The artifact diameter was determined for each of the 4 different coaxial MR-compatible needles for every modification of the scan series. In total, we implemented 2 measurements per needle artifact, which were performed by 2 independent and blinded readers (with 2 and 6 years of diagnostic MR imaging experience) for each modification of the evaluated parameters.

### Statistical analysis

Statistical analysis was performed using dedicated statistics software (SPSS version 26, SPSS Inc., Chicago, IL). For descriptive statistics, the numerical values are presented as mean values plus standard deviation at 95% confidence intervals. To evaluate differences between the modified sequence parameters in the related samples, we used the Wilcoxon signed-rank test in case of 2 values of the modified parameter and in case of > 2 values the Friedman test including post hoc testing and Bonferroni multiple testing correction. Furthermore, we evaluated possible positive or negative correlations between the values of the modified parameters and the size of artifact diameter. For this purpose, we calculated the correlation coefficient Bravais–Pearson, which was tested for significance on both sides. In order to assess the significance of the results, the effect strengths *r* of the correlation coefficient Bravais–Pearson was additionally presented using Cohen's classification (*r* = 0.10 weak effect, *r* = 0.30 medium effect, *r* = 0.50 strong effect). To determine differences between the 4 diameters of MR-compatible intervention needles as unrelated samples, we used the Mann–Whitney U test. To measure the interrater reliability between the 2 blinded readers, we calculated the intraclass correlation coefficient (ICC). For all tests, *p* < 0.05 was considered significant.

## Data Availability

All relevant data are provided in the manuscript.
